# Can postnatal steroids or initial mode of respiratory support really impact lung function at adulthood in preterm survivors?

**DOI:** 10.1038/s41390-025-04391-5

**Published:** 2025-09-17

**Authors:** Shivanthan Shanthikumar, David N. Matlock, David G. Tingay, Sherry E. Courtney

**Affiliations:** 1 Department of Respiratory Medicine, The Royal Children’s Hospital, Parkville, VIC Australia; 2 Department of Paediatrics, University of Melbourne, Melbourne, VIC Australia; 3 Respiratory Diseases, Murdoch Children’s Research Institute, Parkville, VIC Australia; 4 University of Arkansas for Medical Sciences, Little Rock, AR USA; 5 Arkansas Children’s Hospital, Little Rock, AR USA; 6 Neonatal Research, Murdoch Children’s Research Institute, Parkville, VIC Australia; 7 Department of Critical Care, University of Melbourne, Melbourne, VIC Australia

Globally, around 15 millions infants are born prematurely each year and due to improvements in perinatal care most now survive to hospital discharge.^1^ This means that there is a large, and ever-expanding population of children, adolescents and now adults who are “survivors” of prematurity. People born preterm have an increased risk of disorders in multiple systems, including neurological, metabolic, cardiovascular and respiratory, which culminate in increased morbidities and mortality in adulthood.^2^ As such there is an urgent need to identify (1) the exact nature and prevalence of comorbidities encountered from people with a history of prematurity, (2) how these comorbidities can be proactively screened for and managed, and (3) elucidate modifiable early life exposures that are associated with risks of later life complications.

In this issue of *Pediatric Research* Jenkinson and colleagues describe a study protocol that aims to address these knowledge gaps.^3^ They propose to follow up a subset of the original United Kingdom Oscillatory Study (UKOS) cohort, babies who were born at <29 weeks gestation and are now 24–28 years of age, along with age-matched term-born controls. The study aims to enroll 200 participants, 150 from the original UKOS preterm cohort (representing approximately 25% of the survivors). These 150 UKOS participants will be compared to 50 control subjects born at term gestation. Study participants will undergo a comprehensive suite of assessments including questionnaires (to assess demographics, educational/employment outcomes, cognition, quality of life, and mental health), comprehensive lung function testing, echocardiography, and biospecimen collection. The UKOS trial was one of the largest randomized controlled trials comparing first intention high-frequency oscillatory ventilation (HFOV) with conventional mechanical ventilation (CMV) in preterm infants.^4^ The study found no difference in the primary outcome of survival free of BPD, in contrast to a similar study published at the same time which found a small improvement in survival free of BPD in infants receiving HFOV.^5^ The UKOS study was more pragmatic, resulting in less compliance with a high lung volume HFOV strategy compared to the study by Courtney, et al.^6^

The proposed study is primarily focused, and powered to detect differences in spirometric indices of lung function and assess the impact of postnatal corticosteroid exposure on these indices. People born preterm are known to have a higher prevalence of reduced lung function, typically associated with Chronic Obstructive Pulmonary Disease (COPD), which in itself is associated with reduced quality of life, respiratory and cardiovascular morbidity, and increased mortality.^7^ The origins of COPD arise in the early years and likely include antenatal and perinatal insults. In this setting, it is no surprise that prematurely born infants are at greater risk, and it is important to understand which aspects of neonatal care affect the risk of subsequent COPD. Unfortunately, linking COPD to specific early life treatments, when risk can be best modulated, has been lacking. The UKOS cohort already shed some light on this problem. A follow-up study identified that postnatal corticosteroid exposure was associated with increased risk of several markers of obstructive lung function at 11–14 years of age including FEV1, and FEV1/FVC in 179 of the survivors assessed.^8^ This finding persisted at 16–19 years of age, with 60% of corticosteroid exposed participants having an obstructive abnormality compared to 27% in the unexposed group.^9^ Further some indices (FEV1, FEF75) deteriorated in the steroid exposed group during adolescence. Lastly, both studies demonstrated a dose response relationship, with worse lung function observed in participants exposed to repeated corticosteroid courses. The proposed study will expand the contribution to this topic by exploring the relationship between postnatal corticosteroid exposure and subsequent obstructive lung disease and assess whether the association persists to 24–28 years of age (the time in which lifelong lung function peaks). Regarding the ability to determine whether postnatal corticosteroid exposure causes compromised lung function later in life, this study will have the same limitations as the earlier follow up. As opposed to HFOV, administration of postnatal corticosteroids was a secondary outcome of the UKOS trial, not a randomized intervention. Careful statistical efforts at controlling for disease severity are needed as infants with the most severe lung disease would have been more likely to receive postnatal steroids.

To date, in order to assess the long term lung function outcomes of HFOV compared to CMV, the UKOS study has followed up 319 and 159 of the original participants at 11–14 years and 16–19 years of age.^10^ At 11–14 years of age they identified increased obstructive lung disease in the CMV group.^11^ However, there were no differences between the two groups at 16–19 years of age,^12^ and no differences in the longitudinal change between these two assessments.^10^ The lack of difference at 16–19 years of age may be a true result or it may be a false negative finding highlighting the difficulties and interpretation risks in longitudinal cohort studies of preterm infants who are exposed to a multitude of respiratory treatments. The proposed study will reassess this question in early adulthood; however, the relatively small sample size in the proposed study will be a significant limitation.

The primary outcome in the original UKOS study was the rate of BPD or death. The original study found similar rates of BPD between CMV and HFOV, despite the physiological rationale for reduced lung injury using appropriately applied HFOV. Arguably, the greatest value of the UKOS study has been to demonstrate the potential limitations of assessing early respiratory outcomes such as BPD, and the need to follow lung function through to adulthood to ensure lifelong impacts are not missed. Since follow-up at several earlier time points has been performed on many of these infants it would be of great interest to know what subjects in the currently planned study had BPD and how those infants are doing as young adults compared to those who were not diagnosed with BPD. This could be of use in prognosticating for today’s BPD infants.

Long-term follow-up results for the preterm population are always moving targets. These results reflect the care given at the time, and these practices change constantly. Initial respiratory care of preterm infants today consists of significantly more non-invasive support than it did over twenty years ago. The results of the proposed study will clearly define the current respiratory status of infants born preterm many years ago, but we must remember that some results will be due to prematurity, while others may be due to the treatments received in that era. This includes not only HFOV vs CMV, but minimal use of non-invasive ventilation, early extubation, nutritional care and volume-targeted ventilation among others.

With the design of the proposed study, and in particular the sample size, there will be selection bias towards participants who have survived, are able to be contacted, and are able to participate. A CONSORT diagram would be of great interest and might assist others with future long-term follow-up. It would be a missed opportunity not to see how many long term survivors could be contacted and would at least consent to a simple questionnaire as to their respiratory status, as was recently performed in the OPTIMIST study.^13^ Another way to mitigate selection bias would be to review admission data, vital records (death certificates) and ideally health care usage if these are available for those who are unable to participate.

The study will also explore several other outcomes related to long term complications of prematurity, including pulmonary hypertension, exercise tolerance, muscle strength, systemic and airway inflammation, and airway dysbiosis. These outcomes will be assessed via comparison between the premature and term born controls. This comprehensive suite of assessments will better delineate the range of comorbidities experienced by young adults born prematurely, and may inform the development of much needed screening health assessments for this population.

The UKOS study was the only large HFOV trial able to fund and perform follow-up. It should not have been. The possible long-term benefits of HFOV over CMV, and the impacts of early postnatal steroids, are still open questions. We congratulate the investigators on their proposed study and look forward to the results. Irrespective of the outcomes this study will demonstrate the feasibility of building in long-term respiratory follow up into NICU trials and we hope it will not be the last.^14^
